# Prevalence of self-perceived audiovestibular symptoms in Egyptian COVID-19 patients

**DOI:** 10.1186/s42506-023-00143-7

**Published:** 2023-09-18

**Authors:** Mirhan Eldeeb, Dalia Eldeeb, Mayada Elsherif

**Affiliations:** 1 Department of Otorhinolaryngology, Audiovestibular Medicine Unit, Faculty of Medicine, Alexandria University, Al Sultan Hussein Street, Al Khartoum Square, Al Azareeta, Alexandria, 21111 Egypt; 2 Public Health and Community Medicine Department, Faculty of Medicine, Alexandria University, Alexandria, Egypt

**Keywords:** Audiovestibular symptoms, COVID-19, Hearing loss, Vertigo, Tinnitus, Ear fullness

## Abstract

**Background:**

According to National Institute for Health and Care Excellence (NICE), UK, common audiovestibular symptoms of COVID-19 include dizziness, tinnitus, and otalgia. The pathogenesis of otologic disorders ranges from direct damage to the inner ear structures to immune-mediated damage. Since the start of the pandemic, the prevalence of audiovestibular symptoms linked to COVID-19 has not been thoroughly investigated in Egypt. Our objective is to study and analyze the prevalence of the audiovestibular symptoms in the Egyptian population with history of COVID-19 infection.

**Methods:**

A cross-sectional study was conducted among Egyptian adults on the presence and nature of the audiovestibular manifestations in COVID-19 patients. An online questionnaire was used. The questionnaire was developed using Google Form. It was disseminated to the target population through social platforms from October 2021 till February 2022.

**Results:**

Data from 245 respondents were collected through online assessment of a convenient sample. The following de novo audiovestibular symptoms were experienced by the participants: Vertigo 20.8%, hearing loss 13.9%, tinnitus 12.7% and ear fullness 11.4%. No correlation was found between the investigations done (D-Dimer, CT chest) and the audiovestibular symptoms.

**Conclusion:**

Audiovestibular symptoms are fairly common among COVID-19 patients, with higher prevalence, particularly of vertigo, in our study sample compared to the literature. It is recommended that patients with audiovestibular symptoms undergo early testing so that prompt interventions can be taken.

## Introduction

COVID-19 usually generates a wide range of clinical symptoms in the pulmonary and gastrointestinal systems. Nonetheless, as the pandemic advanced, the range of COVID-19 clinical characteristics widened to encompass the nervous system, with symptoms such as headache, anosmia, encephalitis, decreased consciousness and sporadic cases of Guillain–Barre syndrome [1].

Additionally, the presence of audiovestibular manifestations in COVID-19 patients has also been documented in the literature [2] such as tinnitus, hearing loss, dizziness, and vertigo [3]. Other well-known viral diseases have also been linked to similar audiovestibular signs [4]. The clinical picture of COVID-19 ranges from asymptomatic to severe or fatal [5]. The United Kingdom (UK) National Institute for Health and Care Excellence (NICE; 2020) has described a set of definitions for three phases of signs and symptoms: (i) acute, persisting for up to 4 weeks; (ii) ongoing, from 4 to 12 weeks; and (iii) post-COVID syndrome, continuing for more than 12 weeks (the latter two phases are often grouped together and referred to as ‘long COVID’). According to NICE, common audiovestibular symptoms of long COVID include dizziness, tinnitus and otalgia [5]. The pathogenesis of otologic disorders ranges from direct damage to the inner ear structures to immune-mediated damage [4].

Several pathophysiological mechanisms have been suggested behind vestibular affection in COVID-19 patients. The formation of microthrombi in the circulation is known to occur during the COVID-19 infection and could explain the occurrence of Benign Paroxysmal Positional Vertigo (BPPV) after the initial phase of the SARS-COVID-19 infection. Another possible cause of post- COVID BPPV is the prolonged bed rest leading to rupture of the otoconia due to the insufficient movement [6]. One previous study concluded that medications and the inflammatory process could induce decalcification, thus damaging the otoconia and inducing BPPV [7]. COVID patients may suffer from vestibular neuritis (VN) due to the fact that the Angiotensin- converting enzyme 2 (ACE 2) receptors and TMPRSS2 serve as entry points for the COVID-19 to access the vestibule and produce VN [8]. Another theory explaining the VN in COVID-19 is ischemia of the vasa nervorum in addition to demyelination caused by the inflammation [9]. Acute VN in COVID-19 patients was reported in the literature [6, 10, 11]. Also VN could be a result of direct nerve infiltration by the COVID-19 [12]. Milionis et al., [13] linked VN to the inflammatory process and the increase in levels of plasma fibrinogen and C-reactive protein (CRP). To explain the occurrence of hearing loss in COVID-19 patients, one theory is that acute viral infection induces direct damage of inner ear structures or audiovestibular nerve, owing to viral neurotropism, either directly or through an autoimmune-mediated process [4, 14], where ACE-2, cellular receptor for COVID-19 was detected in many sites in both the middle and inner ears [15]. A second theory which was suggested to explain audiovestibular symptoms is vascular pathology due to the observation that many COVID-19 patients show coagulation anomalies [16]. The inner ear has no collateral circulation, and thus is greatly susceptible to slight insults which can influence its blood supply. This accentuates the vulnerability of cochlea and vestibular end organs to thrombosis or hypoxia, therefore contributing to the occurrence of audiovestibular manifestations, predominantly Sudden Sensorineural Hearing Loss (SSNHL) [17].

A possible mechanism for ear fullness in COVID-19 patients is Eustachian tube dysfunction similar to other viral infections in the nasopharynx. Certainly, the ACE-2 receptor, which is the cellular receptor for the COVID-19, was isolated in both the Eustachian tubes and middle ears of mice [15] thus reflecting that these tissues are probably liable to COVID-19 infection.

Since the start of the pandemic, the prevalence of audiovestibular symptoms linked to COVID-19 has not been thoroughly investigated in Egypt. Our objective is to estimate and analyze the prevalence of the audiovestibular symptoms in the Egyptian population with history of COVID-19 infection.

## Methods

### Study design

A cross-sectional study was conducted from December 2021 till February 2022 among Egyptian adults.

### Data collection

A self-administered questionnaire was developed and included questions about self-reported audiovestibular manifestations among those infected with COVID-19. An online survey portal, Google Form was created, and participants were invited to complete and submit the form.

An introductory paragraph describing the objective of the study was shared with the respondents through social platforms commonly used by Egyptian population on Facebook, Messenger and WhatsApp.

### Sampling

Sample size: Considering the prevalence of tinnitus among adults following COVID 19 infection is 14.8% (AlJasser et al., [18]) and assuming 95% confidence interval, the minimum required sample size calculated using Epi Info (version 7) was 194 subjects. The actual sample size collected was 245 subjects.

Sample type: A convenient sample was used.

### Inclusion criteria

The respondents belonged to different social categories, such as university faculty members, doctors, nurses, engineers, students and housewives. The answers to the survey questionnaire were voluntary. Subjects were instructed to answer the questionnaire if they had COVID-19 infection confirmed by PCR.

The online questionnaire was divided into sections, section one included the sociodemographic data (age, gender, residence, education and occupation). Section two included questions about the frequency and duration of vertigo, ear fullness, tinnitus and hearing loss. Section three included questions about investigations done for patients as CT chest and D dimer tests (for health professionals only).

### Statistical analysis

Data were fed to the computer and analyzed using IBM SPSS software package version 20.0. (Armonk, NY: IBM Corp). The Kolmogorov–Smirnov was used to verify the normality of distribution of variables. Comparisons between groups for categorical variables were assessed using Chi-square test (Monte Carlo). McNemar and Marginal Homogeneity Test were used to analyze the significance between pre and post COVID-19 infection. Wilcoxon signed ranks test was used for abnormally distributed quantitative variables, to compare between two periods. Spearman coefficient was used to correlate between quantitative variables. Significance of the obtained results was judged at the 5% level.

## Results

The questionnaire was distributed for 3 months, and 245 participants responded to the questionnaire. Participants were mostly from urban zone while 19 participants (7.8%) only were from rural zones of Egypt. The highest percentage of participants had a higher education and were professional workers (58%), while 35.5% of them were unemployed and 16 participants did written and handy work. Of the participants, 31.0% were males and 69.0% were females. Participants were mostly in the age group between 18–25 years old (39.6%) and 35–45 years old (33.9%) (Table 1).

**Table 1:** Socio-demographic characteristics of the studied Egyptian adults, 2021–2022 (*n* = 245)

Demographic data	No. (%)
**Gender**	
Male	76 (31.0)
Female	169 (69.0)
**Age in years**	
18- < 25	97 (39.6)
25- < 35	47 (19.2)
35- < 45	83 (33.9)
45–60	12 (4.9)
Above 60	6 (2.4)
**Residence**	
Urban	226 (92.2)
Rural	19 (7.8
**Level of education**	
University	244 (99.6)
High school	1 (0.4)
**Occupation**	
Unemployed	87 (35.5)
Handy work	3 (1.2)
Professional work	142 (58.0)
Written work (Semi-professional)	13 (5.3)

### Occurrence of audio vestibular symptoms among participants

**Fig. 1:**
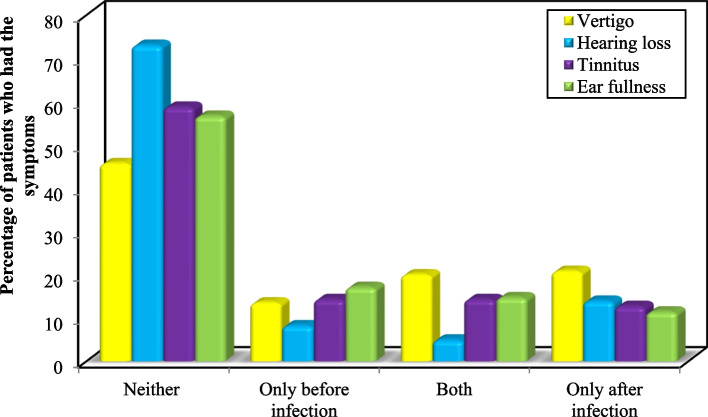
Occurrence of audiovestibular symptoms among the studied Egyptian adults, 2022 (*n* = 245). (Neither: neither before nor after COVID-19 infection, Both: both before and after COVID-19 infection)

Most participants did not experience audiovestibular symptoms neither before nor after COVID-19 infection. Figure 1 shows the distributions of participants according to the onset of audiovestibular symptoms.

With the exception of vertigo, less than 20% of participants experienced audiovestibular symptoms de novo following the COVID-19 infection (Table 2).

**Table 2:** Frequency and characteristics of the audiovestibular symptoms experienced by the studied Egyptian adults, 2022

Vertigo	No. (%)
**Have you ever felt dizzy (feeling like you are spinning or the world is spinning around you) before you contracted COVID-19?**	
No	163 (66.5)
Yes	82 (33.5)
**Did you feel dizzy (feeling like you are spinning or the world is spinning around you) while you were infected with COVID-19 or after you recovered?**	
No	145 (59.2)
Yes	100 (40.8)
**(McN) p**	**3.440 (0.064)**
**If yes, when did you feel dizzy (feeling like you're spinning or the world is spinning around you) while you were infected with COVID-19?**	***n*** = 100)
First week from initial presentation	62 (62.0)
Second week from initial presentation	17 (17.0)
Third week from initial presentation	7 (7.0)
Fourth week from initial presentation	14 (14.0)
**How many times did you feel dizzy while you were infected with COVID-19?**	***(n*** = 100)
Once	21 (21.0)
Twice or more	79 (79.0)
**In case of once, did you feel dizzy following a change in head position, such as leaning forward, looking up, turning over in bed while sleeping, or lying down?**	***(n*** = 21)
No	12 (57.1)
Yes	9 (42.9)
**In case of twice or more, did you feel dizzy following a change in head position, such as leaning forward, looking up, turning over in bed while sleeping, or lying down?**	***(n*** = 79)
No	28 (35.4)
Yes	51 (64.6)
**How long did the vertigo attack last?**	***(n*** = 100)
Seconds	54 (54.0)
Minutes	34 (34.0)
Hours	9 (9.0)
Days	3 (3.0)
**Neither**	**112 (45.7)**
**Only before infection**	**33 (13.5)**
**Both**	**49 (20.0)**
**Only after infection**	**51 (20.8)**
**Hearing loss**	**No. (%)**
**Have you ever experienced hearing loss in one or both ears before you contracted COVID-19?**	
No	213 (86.9)
Yes	32 (13.1)
**Did you experience hearing loss in one or both ears during your infection with COVID-19 or after you recovered?**	
No	199 (81.2)
Yes	46 (18.8)
**(McN) p**	**3.130 (0.077)**
**If yes, was the hearing loss in:**	**(*n* = 46)**
One ear	28 (60.9)
Both ears	18 (39.1)
**When did you experience hearing loss in one or both ears when you were infected with COVID-19?**	**(*n* = 46)**
First week from initial presentation	15 (32.6)
Second week from initial presentation	12 (26.1)
Third week from initial presentation	10 (21.7)
Fourth week from initial presentation	9 (19.6)
**If you experienced hearing loss during COVID-19 infection, was the hearing loss:**	**(*n* = 46)**
Intermittent	35 (76.1)
Continuous	11 (23.9)
**Neither**	**179 (73.1)**
**Only before infection**	**20 (8.2)**
**Both**	**12 (4.9)**
**Only after infection**	**34 (13.9)**
**Tinnitus**	**No. (%)**
**Have you ever heard a buzzing sound or a whistle in your ears before you contracted COVID-19?**	
No	175 (71.4)
Yes	70 (28.6)
**Did you hear a buzzing sound or a whistle in your ears while you were infected with ** **COVID-19 or after you recovered?**	
No	179 (73.1)
Yes	66 (26.9)
**(McN) p**	**0.136 (0.712)**
**If yes, when did you hear a buzzing sound or a whistle when you contracted COVID-19?**	**(*n* = 66)**
First week from initial presentation	27 (40.9)
Second week from initial presentation	13 (19.7)
Third week from initial presentation	11 (16.7)
Fourth week from initial presentation	15 (22.7)
**Did you hear the buzzing sound or the whistle in:**	**(*n* = 66)**
One ear	38 (57.6)
Both ears	28 (42.4)
**How long did the buzzing sound or whistle last during COVID-19 infection?**	**(*n* = 66)**
Seconds	27 (40.9)
Minutes	16 (24.2)
Hours	9 (13.6)
Days	14 (21.2)
**If you heard a buzzing sound or a whistle when you were infected with COVID-19, was the sound?**	**(*n* = 66)**
Intermittent	41 (62.1)
Continuous	25 (37.9)
**Neither**	**144 (58.8)**
**Only before infection**	**35 (14.3)**
**Both**	**35 (14.3)**
**Only after infection**	**31 (12.7)**
**Ear fullness**	**No. (%)**
**Have you ever experienced ear fullness in one or both ears before you contracted COVID-19?**	
No	167 (68.2)
Yes	78 (31.8)
**Did you experience ear fullness in one or both ears during your infection with COVID-19 or after you recovered?**	
No	181 (73.9)
Yes	64 (26.1)
**(McN) p**	**2.414 (0.120)**
**If yes, was the sense of ear fullness in:**	***(n*** = 64)
One ear	33 (51.6)
Both ears	31 (48.4)
**When did you experience ear fullness in one or both ears while you were infected with COVID-19?**	***(n*** = 64)
First week from initial presentation	30 (46.9)
Second week from initial presentation	19 (29.7)
Third week from initial presentation	8 (12.5)
Fourth week from initial presentation	7 (10.9)
**Does the feeling of ear fullness go away after swallowing, pushing hard (as occurs when defecating), yawning or pressing on the tragus (protrusion in the frontal part of the external ear opening)(an illustration was provided)?**	***(n*** = 64)
No	32 (50.0)
Yes	32 (50.0)
**Neither**	**139 (56.7)**
**Only before infection**	**42 (17.1)**
**Both**	**36 (14.7)**
**Only after infection**	**28 (11.4)**

*McN* McNemar test, *p* p value for comparing between pre and post

(Neither: neither before nor after COVID-19 infection, Both: both before and after COVID-19 infection)

^*^Statistically significant at *p* ≤ 0.05

Table 3 shows the distribution of the questionnaire respondents according to the number of audiovestibular symptoms they experienced before and after COVID-19 infection (*n* = 245). The mean number of audiovestibular symptoms was 1.07 ± 1.28 before COVID-19 infection compared to 1.13 ± 1.13 post infection and the difference was not statistically significant (*p* = 0.562).

**Table 3:** The number of audiovestibular symptoms reported by the studied adults in Egypt, 2022 (*n* = 245)

Number of symptoms	Pre	Post	Test of sig	*P*
	**No. (%)**	**No. (%)**		
0	116 (47.3)	106 (43.3)	MH = 198.0	0.562
1	55 (22.4)	64 (26.1)		
2	32 (13.1)	30 (12.2)		
3	25 (10.2)	28 (11.4)		
4	17 (6.9)	17 (6.9)		
Mean ± SD	1.07 ± 1.28	1.13 ± 1.13	Z = 0.624	0.533
Median (Min – max.)	1.0 (0.0 – 4.0)	1.0 (0.0 – 4.0)		

*MH* Marginal Homogeneity Test. Z: Wilcoxon signed ranks test, *p* p value for comparing between pre and post, *No. symptoms* Number of symptoms, Pre: before COVID-19 infection, Post: after COVID-19 infection

^*^Statistically significant at *p* ≤ 0.05

### D-dimer and CT chest

Health professionals were advised to report either the outcome of these investigations or the fact that they did not take the test, while other subjects were instructed to choose the option "not a health care professional" when answering questions about these investigations.

**Fig. 2:**
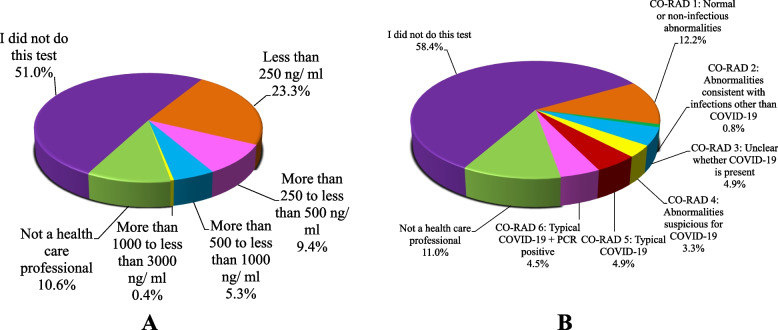
The results of the investigations reported by the respondents: **A** Distribution of the respondents according to the results of D-Dimer test (*n* = 245). **B** Distribution of the respondents according to the results of the Chest CT scan (*n* = 245)

Figure 2 shows the results of the investigations reported in all the participants included in the study. More than half of the respondents reported not doing either test, D-dimer (51%) and chest CT scan (58.4%). The Chi square and Monte Carlo tests did not show statistical significance between the level of D-dimer (*n* = 94) (38.3%), CT chest findings (*n* = 75) (30.6%) and the audiovestibular symptoms (vertigo, hearing loss, tinnitus, and ear fullness) (*p* > 0.05).

The correlation between the D-dimer (*n* = 94, 38.3%), CT chest findings (*n* = 75, 30.6%) and subjects’ replies to the questions about audiovestibular symptoms showed statistical significance (Table 4).

**Table 4:** Correlation between investigations’ results and the participants’ reported symptoms of Vertigo, Hearing loss, Tinnitus and Ear fullness

Symptoms	D-Dimer test (This question was for health care professionals only) (*n* = 94)		COVID-19 Reporting and Data System (CO-RADS) (this question was for health care professionals only) (*n* = 75)	
	**r**_**s**_	***p***	**r**_**s**_	***p***
**Vertigo**				
When did you feel dizzy (feeling like you're spinning or the world is spinning around you) while you were infected with COVID-19?	0.340^*^	0.001^*^	0.074	0.462
How many times did you feel dizzy while you were infected with COVID-19?	-0.058	0.564	0.118	0.242
How long did the vertigo attack last?	-0.080	0.428	-0.073	0.470
**Hearing loss**				
Was the hearing loss in one or both ears	-0.165	0.273	-0.168	0.264
When did you experience hearing loss in one or both ears when you were infected with COVID-19?	-0.020	0.896	0.205	0.171
If you experienced hearing loss during COVID-19 infection, was the hearing loss intermittent or continuous	0.249	0.096	-0.103	0.496
**Tinnitus**				
If yes, when did you hear a buzzing sound or a whistle when you contracted COVID-19?	0.195	0.116	0.123	0.325
How long did the buzzing sound or whistle last during COVID-19 infection?	-0.003	0.978	0.178	0.153
if you heard a buzzing sound or a whistle when you were infected with covid-19, was the sound intermittent or continuous?	0.067	0.593	0.092	0.462
**Ear fullness**				
Was the sense of ear fullness in one or both ears?	-0.008	0.950	0.028	0.828
When did you experience ear fullness in one or both ears while you were infected with COVID-19?	0.078	0.539	0.020	0.876
**No. symptoms (pre)**	-0.015	0.815	-0.024	0.707
**No. symptoms (post)**	0.147^*^	0.021^*^	0.155^*^	0.015^*^

r_s_ Spearman coefficient

^*^Statistically significant at *p* ≤ 0.05

No. symptoms (pre): number of symptoms before COVID-19 infection

No. symptoms (post): number of symptoms after COVID-19 infection

## Discussion

We utilized a questionnaire to describe the audiovestibular symptoms in post COVID-19 patients, as well as their demographic features, and the results of their investigations in relation to cochleovestibular manifestations. Overall, the current study found that audiovestibular affection was widespread in the initial phase of COVID-19 infection. During the first four weeks following the first symptom, newly acquired vertigo was the most frequently reported cochleovestibular complaint (20.8%). Patients also reported de novo hearing loss (13.9%), tinnitus (12.7%) and aural fullness (11.4%) with all the symptoms being mostly reported during the first week following onset of the disease (vertigo 62.0%, ear fullness 46.9%, tinnitus 40.9% and hearing loss 32.6%).

Of our patients, 100 (40.8%) experienced vertigo during or after their COVID 19 infection with 21% of them experienced vertigo once, while the rest had vertigo twice or more. Of the patients who experienced vertigo following the infection, 57.1% experienced spontaneous vertigo whereas the rest had positional vertigo. Fifty four percent had vertigo for seconds, 34% for minutes and 9% for hours. Three percent of our sample experienced vertigo for days which could denote an inflammation in the vestibular nerve or vestibular neuritis (VN) [8]. Forty six percent of our sample experienced vertigo similar to Meniere’s disease (ranging from minutes to hours), which could be explained by the immunological theory described by Bumm et al., [12], where T4 T-helper and T8 T-suppressor cells were found in inner ear diseases (e.g., Menière’s disease, sudden hearing loss, vestibular neuritis and Bell’s Palsy) using specific monoclonal antibodies. In a previous study done in 2020, 18.4% of patients reported equilibrium disorders after they were positively diagnosed with COVID-19, where 94.1% of them reported dizziness and 5.9% reported acute attacks of vertigo [19]. The lower percentage of vertigo reported by Viola et al., [19] is probably because the authors did not explain the nature of vertigo to patients (spinning sensation of self or surroundings), which we stressed in our questionnaire to differentiate between vertigo and other balance disorders.

The estimated prevalence of newly acquired hearing loss (13.9%) in this study is higher than a recent study by Almishaal et al., who reported a prevalence of 6.31% in their study, which utilized a questionnaire to assess the short-term and long-term cochleovestibular symptoms following COVID-19 infection in 301 severe hospitalized and mild non-hospitalized patients [20]. Conversely, another study used hearing handicap inventory during convalescence period, and found that hearing loss appeared or worsened in 40% of patients [21]. These differences may be related to differences in the methods and tools used for assessment of symptoms and self-reporting may be associated with over or under reporting.

The prevalence of tinnitus in this study was found to be 12.7% (57.6% unilateral and 42.4% bilateral), which is, yet again, higher than Almishaal et al. (9.97%) [20]. Nevertheless, the frequency of tinnitus as a symptom in case studies of COVID-19 patients seems to be much higher, where a systematic review of auditory disturbances in COVID-19 patients [22] reported four out of 16 patients (25%) (two unilateral and two bilateral) complaining of tinnitus. Conversely Gallus et al., [23] investigated audiovestibular affection in 48 post COVID-19 patients in a retrospective study using pure-tone audiometry thresholds, tympanometry, and Stapedius reflex, and only two patients (4.2%) reported tinnitus. Surprisingly, all the investigations showed normal results and the authors concluded that the symptoms were transient and no permanent audiovestibular damage has occurred.

Ear fullness was reported in 11.4% of our patients which is less than that reported in Almishaal et al., (18.94%) [20]. Similar frequencies of ear fullness in COVID-19 patients were found in previous studies [24, 25].

Presence of the virus in the middle ear and Eustachian tube [15, 26] could explain the intermittent hearing loss and ear fullness experienced by our patients owing to middle ear infection and Eustachian tube dysfunction.

No significant difference was found between study groups and the level of D-dimer (*n* = 94), however a moderate significantly positive correlation (r_s_ = 0.340, *p* = 0.001) was found between patients who reported vertigo following COVID-19 infection and the level of their D-dimer, as well as a weak significantly positive correlation between the number of audiovestibular symptoms and the level of D-dimer (r_s_ = 0.147, *p* = 0.021). D-dimer has been used as an efficient biomarker of thrombotic tendency in COVID-19 patients and has been associated with higher disease severity [27]. The presence of vertigo in COVID-19 patients together with elevated D-dimer levels has been reported in the literature [16]. The formation of microthrombi in the circulation is known to occur during the COVID-19 infection and could explain the occurrence of Benign Paroxysmal Positional Vertigo (BPPV) after the initial phase of the SARS-COVID-19 infection. This might explain the positional vertigo, which was reported in 42.9% of our sample [7]. This theory is supported by our results, which showed a positive correlation between the D-dimer and vertigo (*r* = 0.340, *p* = 0.001).

The COVID-19 Reporting and Data System (CO-RADS) can be used, along with certain biochemical markers, as a predictor of ICU admission (94.2% accuracy) in COVID-19 subjects, and therefore is a good indicator of disease severity [28]. When different study groups were compared in terms of CO-RADS grading system, no significant difference was found, nonetheless a weak significantly positive correlation between the number of audiovestibular symptoms and the CO-RADS score (*n* = 75) (r_s_ = 0.155, *p* = 0.015).

### Limitations

This study has some limitations. First, COVID-19 severity was not assessed among the survey respondents. Consequently, the severity of the condition could not be linked to the occurrence of audiovestibular symptoms. Second, the patients were not clinically examined yet, our study examines de novo audiovestibular symptoms. Third, the study used online reporting of subjective symptoms which might be affected by other factors such as the recall bias and mental state of the patient.

## Conclusion

Audiovestibular symptoms are fairly common among COVID-19 patients, with higher prevalence, particularly vertigo, in our study sample compared to the literature. The following de novo audiovestibular symptoms were experienced by the respondents: Vertigo 20.8%, hearing loss 13.9%, tinnitus 12.7% and ear fullness 11.4%. It is recommended that patients with audiovestibular symptoms undergo early testing (audiometry, vestibular examination…..etc.) so that prompt interventions can be taken.

## Data Availability

Data are available from the corresponding author on reasonable request. Confidentiality and security of data and materials were ensured through all stages of the study.

## Abbreviations

ABR: Auditory Brainstem Response
ACE-2: Angiotensin Converting Enzyme 2
BPPV: Benign Paroxysmal Positional Vertigo
CMV: Cytomegalovirus
CO-RADS: The COVID-19 Reporting and Data System
COVID-19: Corona Virus Disease of 2019
CRP: C-reactive protein
CT: Computerized Tomography
NICE: National Institute for Health and Care Excellence
PCR: Polymerase Chain Reaction
SARS-CoV-2: Severe Acute Respiratory Syndrome Corona Virus 2
SNHL: Sensorineural hearing loss
SSNHL: Sudden Sensorineural Hearing Loss
TEOAE: Transient Evoked Otoacoustic Emissions
TMPRSS2: Transmembrane Protease, Serine 2
VN: Vestibular Neuritis
